# Challenges of Health Surveillance today

**DOI:** 10.1590/S2237-96222022000200017

**Published:** 2022-06-27

**Authors:** Carmen Fontes de Souza Teixeira

**Affiliations:** 1 Universidade Federal da Bahia, Instituto de Saúde Coletiva, Salvador, BA, Brazil

Addressing the COVID-19 pandemic has intensified the debate about the possibilities and limits of surveillance actions and practices in the field of health. In this sense, the World Health Organization (WHO) and other international organizations have been discussing the need to structure policies and actions to address health crises, suggesting that surveillance should be treated using a comprehensive approach and as part of prospective risk management, rather than sectoral approaches and management in reaction to harm and consequences.^1^

In Brazil, the debate on strategies to address the pandemic has not only reactivated a conceptual reflection on the notion of health surveillance, present ever since the Brazilian National Health System (*Sistema Único de Saúde* - SUS) began to be built; it has also stimulated the formulation of proposals by the Collective Health (*Saúde Coletiva*) academic community, such as the Front for Life Plan (*Plano da Frente pela Vida*),^2^ and has reactivated a discussion led by the National Health Council in 2018, during the 1^st^ National Health Surveillance Conference,^3^ which was a landmark in preparing the National Policy on Health Surveillance.^4^

Analysis of the conceptual development of health surveillance shows that this notion is linked to the distinct forms of “health surveillance” - epidemiological, environmental, occupational health, etc. - denominating institutional practices that delimit objects, methods and technologies of intervention in health problems and population groups exposed to specific risks. However, the same notion, whether as surveillance for, from or in health, has also contributed to the debate on the change of the care model, as

[...] a form of organization of a heterogeneous set of health practices - health promotion, risk and disease prevention, care and rehabilitation -, in view of the principle of comprehensive care and the possibility of integrating these various practices in the process of reorganizing health work at the different organizational levels of the system.^5^

The process of institutionalizing health surveillance, in turn, has taken place under the auspices of a fragmented view of health surveillance, showing uneven development in recent decades, both with regard to the definition of legislation, which underlies and regulates surveillance actions, and also in the political-institutional sphere, due to the directional nature given to health policy and to the process of building the SUS.^6^ As such, national management of health surveillance has become divided between the Ministry of Health and the National Health Surveillance Agency (*Agência Nacional de Vigilância Sanitária* - ANVISA), while implementation of the different forms of "health surveillance" at the state and municipal levels of the SUS has been marked by tensions and conflicts over funding and forms of organization and the division of management of health surveillance actions between the State and Municipal Health Departments.^7^

Nevertheless, it is worth highlighting the efforts to articulate primary care and health surveillance activities, the management of which is institutionally separate, in both the Ministry of Health and the State Health Departments’ organization charts. Despite this, during the period in which the Family Health Strategy was expanded, the proposal was made to go beyond care based on spontaneous demand, which predominates in primary healthcare centers, by training health teams to plan and program actions based on the analysis and prioritization of the health problems of the population in the territories covered by health centers. This therefore sought to articulate practices related to health promotion, risk prevention and care for prioritized population groups,^8^ ^,^ ^9^ so as to get closer to the comprehensive conception of health surveillance, which seeks to articulate the control of determinants, risks and harm to the health of the population in the different territories.^5^

In recent years, much of this has been lost, not only because of the difficulties that the SUS has suffered over the course of its history, but above all as a result of the changes made by the federal government with effect from 2015, as well as the accelerated process of dismantling health policies and programs.^10^ Indeed, underfunding, privatization, precarization of work and the drastic reduction in investments in infrastructure, science and technology, personnel training and qualification and, specifically, the political decisions regarding primary care funding and organization,^11^ have intensified the structural problems faced by the SUS, which, added to the outbreak of the COVID-19 pandemic, has called into question the viability of maintaining the health system as proposed in the 1988 Federal Constitution.^12^ ^,^ ^13^ Over the course of the pandemic, despite the countless problems that characterized federal management of the crisis, state governors and city mayors implemented measures for prevention, transmission control and care for severe cases by expanding the number of beds, hiring personnel and purchasing equipment, rallying laboratories and research centers, adopting mobility restriction measures and providing economic support to a significant part of the population facing unemployment and a drop in their family income.^14^ In turn, primary care faced serious problems due to lack of knowledge about COVID-19 and precarious health team working conditions, so that in many cases there was a drastic reduction in the number of individuals seen to, with pent-up demand for care for high-incidence chronic diseases.^1^ ^,^ ^15^ Notwithstanding, many municipalities took steps to adapt their infrastructure, train and protect health professionals, including a huge effort to implement the vaccination program, when vaccines became available.

In this context, in the discourse and practice of health institutions, the coexistence of a restricted conception of surveillance can be seen, limited to disclosure of information about the number of cases, deaths and occupancy rates of general ward beds and intensive care unit beds, along with recommendations for "social distancing", "quarantine", care protocols for people with COVID-19 and, finally, vaccination, while the discourses of specialists and scientific entities in the area of Collective Health point to an expanded conception of health surveillance, recognizing the need for articulation between the different surveillance services - cross-cutting the various SUS levels of care - and health promotion actions and primary care at the territorial level, promoting, furthermore, social support measures for vulnerable groups, besides combating fake news.

The debate around the COVID-19 pandemic has reproduced the different theoretical and ideological stances that cross the field of Collective Health, with the appearance of discourses and practices that show the permanence of the "old" view of public health, subordinated to the medical-hegemonic model. Moreover, this model has been strengthened by the expansion of hospital care characterized by private management of public services, compared to "new" public health proposals, based on evidence that guides the definition of strategies and actions focused on specific groups.^16^

Meanwhile, the critical side of the scientific community in the area, also represented in the group that prepared the Front for Life Plan, has pointed out the complexity of the pandemic,^17^ highlighting the context of great economic and social inequality that favored its spread in Brazil. They have insisted on the need for comprehensive action, based on the concept of "social determinants of health",^18^ ^,^ ^19^ articulating intersectoral policies on health promotion and improvement of the quality of life of the population, with specific proposals aimed at priority social groups, due to their greater social vulnerability, as well as the defense of an integrated, free and universal health system, guaranteed by the Constitution, and widespread diffusion of communication and health education actions, aiming at raising the population’s health awareness, based on the understanding that this is a necessary condition for addressing the current and future epidemics.

As I pointed out elsewhere,^20^ the COVID-19 pandemic has made evident the paradigmatic tensions present in the field of health and in the field of Collective Health in particular, intensifying the theoretical and political conflicts between i) the biomedical, clinical and hospital-centric perspective, ii) the epidemiological perspective, which underlies the practices of the "old" and the "new" public health and iii) the radically critical Collective Health perspective, based on the conception of the pandemic as a "hyper-complex" phenomenon, demanding interdisciplinary studies capable of dealing with its multiple dimensions - determinants, characteristics and effects on the population’s health and living conditions. At the same time, the emergence of COVID-19 proposes the implementation of policies and action plans that focus on the various dimensions of a pandemic.

Therefore, the production of knowledge and the development of technologies are challenges for the area, as is the improvement of health surveillance policies and practices based on the integral, intersectoral and participatory conception, as focused by researchers, health service managers and political leaders committed to the Brazilian Health Reform. This requires the unleashing of processes of change, in a context of uncertainty about the country’s economic and political situation in the coming years. However, one can think of health surveillance as a proposal for action capable of inspiring and guiding decision-making about "what to do" in each concrete reality, with a view to resuming the process of building the SUS.
